# Mini-review: advances in 3D bioprinting of vascularized constructs

**DOI:** 10.1186/s13062-020-00273-4

**Published:** 2020-11-02

**Authors:** Lorenzo Bova, Fabrizio Billi, Elisa Cimetta

**Affiliations:** 1 Department of Industrial Engineering (DII), University of Padua, Via Marzolo 9, 35131 Padova, Italy; 2 UCLA Department of Orthopaedic Surgery, David Geffen School of Medicine, Los Angeles, CA USA; 3 Fondazione Istituto di Ricerca Pediatrica Città della Speranza (IRP) - Corso Stati Uniti 4, 35127 Padova, Italy

**Keywords:** Bioprinting, Vascularization, 3D models

## Abstract

3D in vitro constructs have gained more and more relevance in tissue engineering and in cancer-modeling. In recent years, with the development of thicker and more physiologically relevant tissue patches, the integration of a vascular network has become pivotal, both for sustaining the construct in vitro and to help the integration with the host tissue once implanted. Since 3D bioprinting is rising to be one of the most versatile methods to create vascularized constructs, we here briefly review the most promising advances in bioprinting techniques.

## Background

Almost 30 years after Langer [1] introduced the concept of tissue engineering, the field has seen huge progress; in vitro-created tissue patches and organs have already reached clinical use, albeit many challenges are yet to be overcome. In parallel with tissue engineering, 3D in vitro models are also invaluable in cancer research [2]. It is known that the microenvironment plays a crucial role in tumor growth and development, both at the biochemical and biophysical level (structural and soluble component, respectively). Mathematical models strive to capture this complexity and would greatly benefit from the development of novel in vitro systems that better recapitulate the true in vivo behavior of tumors, thus allowing proper model validation and parameter tuning [3]. Metastatic disease is still the cause for > 90% of all cancer-related deaths, relying on processes such as epithelial-mesenchymal transition (EMT) [4], extravasation of stem-like cells from the tumor mass [5], and dependence on new vessels formation [6]. Once again, key unknown regulating these phenomena can find answers thanks to the adoption of better in vitro models.

3D bioprinting has risen as one of the most promising engineering techniques to manufacture in vitro tissues, paving the way to create thick cell-laden constructs [7, 8]. Howbeit, with thickness comes a new hurdle: the need for vasculature to provide nutrients to the cells in the bulk. Creating vascularized tissue constructs further increased the complexity of in vitro tissues, requiring refined 3D bioprinting techniques, more advanced materials and optimized biological protocols. Several different printing methods fall under the bioprinting category, but only a few have been effectively employed to obtain vascularized constructs.

### Extrusion bioprinting: sacrificial inks

**Fig. 1:**
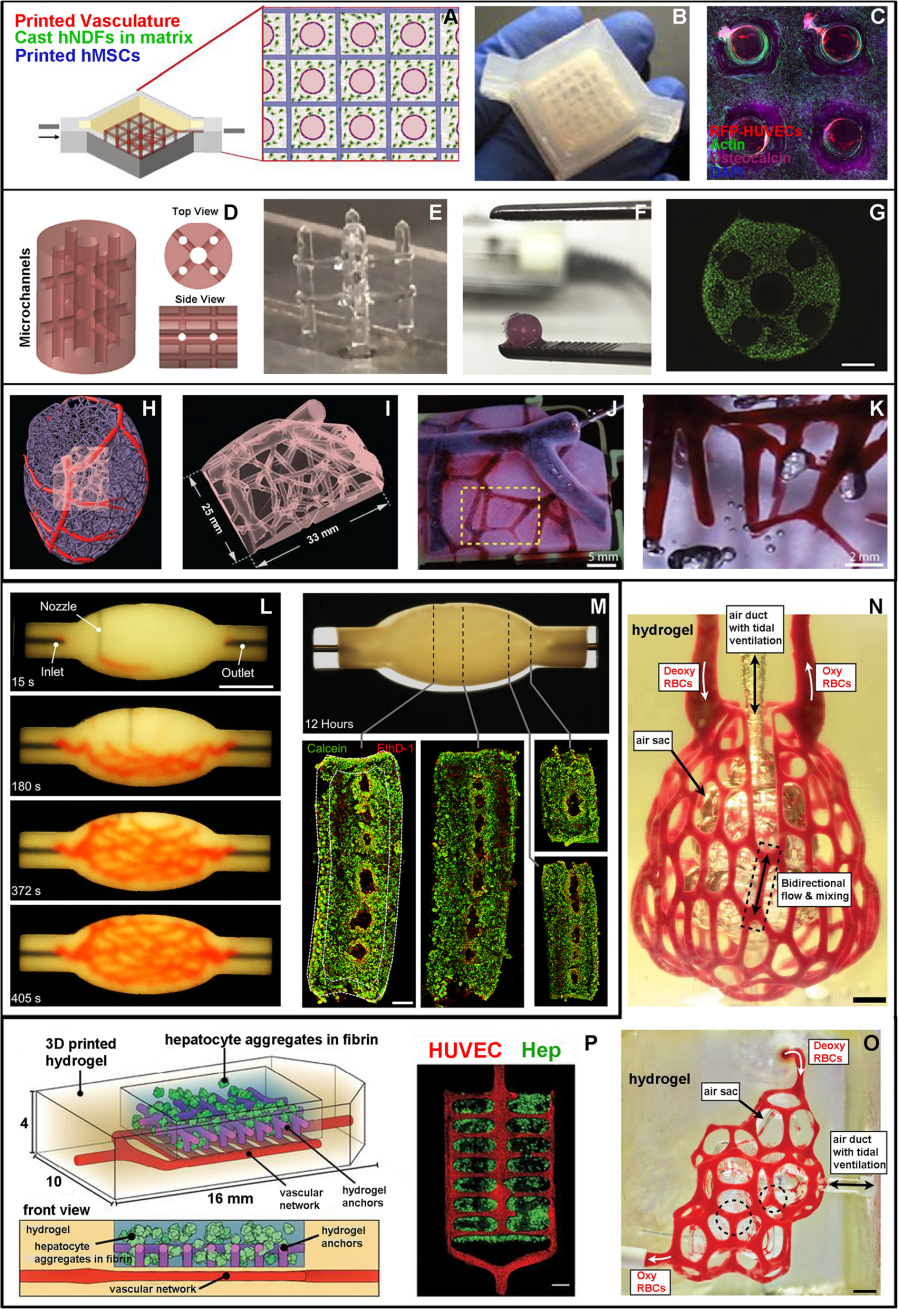
**a**-**c** “Thick perfusable construct” (adapted from Kolesky et al., 2016). **a** Schematic representation of the printed tissue composed of: i. sacrificial ink to fabricate the vasculature network (red circles), ii. human mesenchymal stem cell (hMSC) laden hydrogel (blue squares), and iii. The surrounding hydrogel containing human neonatal dermal fibroblasts (hNDF, green dots); **b** picture of the final bioreactor containing the tissue construct; **c** confocal image of a cross-section after 30 days of perfusion; the construct is densely populated by viable cells (hMSCs, DAPI and actin marking nuclei and cytoskeleton) with a higher osteocalcin expression the closer they are to the channels; HUVECs surrounding the internal cavity of the channels are also visible. **d**-**g** “Implantable vascularized construct for bone repair” (adapted from Daly et al., 2018). **d** Schematic representation of the vascularized construct; **e** freestanding filament network printed with PLU; **f** final GelMA construct after PLU wash out; **g** fluorescence image showing live/dead (green/red) MSCs 24 h after fabrication, scale bar 500 μm. H-K. “Multi-scale MRI-derived vascular network” (adapted from Lee et al., 2019). **h** Computational representation of left ventricle vasculature; **i** subregion chosen for 3D bioprinting; **j** perfusion of the final structure with magnified detail in **k**. **l**-**m** “Branched vascular network printed in a spheroid support bath” (adapted from Skylar-Scott et al., 2019) **l** images at different timepoints during printing of the branching vascular network in a spheroid-based matrix, scale bar 10 mm; **m** fluorescence images at different sections (dashed lines) after 12 h of perfusion, showing live/dead (green/red) iPSCs forming the spheroids. **n**-**p** “Vascularized alveolar and hepatic tissue models” (adapted from Grigoryan et al., 2019). **n**, **o** Printed alveolar models, characterized by a central air sac surrounded by a blood perfused vascular network, scale bars 1 mm; **p** schematic representation and fluorescence imaging of a hydrogel loaded with hepatic cells (green) and supported by a vascular network (red)

Extrusion bioprinting is the cheapest and most versatile technique, not only allowing customization from the engineering perspective (e.g. multi-printheads, coaxial nozzles, microfluidic extrusion) [9–11] but also showing great flexibility in terms of materials choice. Sacrificial inks are fundamental for the successful bioprinting of vascularized constructs: they must enable extrusion in air or in a support bath, and to be liquified and washed out at some later time, leaving a hollow structure inside a different surrounding material [12]. Sacrificial inks include Pluronic F-127 (PLU) [7, 12–14], carbohydrate-glass [15], gelatin [16–18], and agarose [19]. As an example, *Kolesky* et al. used PLU to create a series of hollow channels inside a gelatin methacrylate (GelMA) chip; these channels were then seeded with human umbilical vein endothelial cell (HUVEC), obtaining perfusable endothelialized vascular channels [13]. In a later work by the same group [7], perfusable constructs with a thickness > 1 cm were realized, showing cell culture and survival for up to 6 weeks, and demonstrating that PLU can be successfully printed in air creating stable freestanding structures with pillars and bridges (Fig. 1a-c). Similarly, *Daly* et al. created a vascularized construct printing a sacrificial 3D PLU freestanding structure and surrounding it with cell-laden GelMA (Fig. 1d-g); the construct was implanted in a femoral defect in a rat model, showing a higher host osteoclasts and immune cells invasion compared to a non-vascularized construct [20].

## Extrusion bioprinting: support baths

Although these approaches succeeded in obtaining vascularized tissue patches, they are usually constrained to channel diameters > 200–300 μm and to simple geometries to ensure the stability of the structure. In an effort to overcome such limitations, several studies focused on the optimization of the support bath material, allowing extrusion of more complex 3D vascular networks. *Highley* et al. developed a self-healing bath based on modified hyaluronic acid which deforms when the nozzle is inserted but quickly heals around the printed material [21]. *Bhattacharjee* et al. used a Carbopol ETD 2020 granular support gel to print channels with features and internal diameters < 100 μm [22]. *Hinton* et al. proposed their freeform reversible embedding of suspended hydrogels (FRESH) technique, and developed a slurry containing gelatin microparticles, enabling printing of hydrogels such as alginate, collagen type I, Matrigel and fibrin, which can subsequently be liquified and removed [23]. By bioprinting branched vascular networks and scaled-down models of heart and brain, this study paved the way to their more recent work [24] where the gelatin microparticles diameter was decreased down to ~ 25 μm while maintaining the polydispersity and sphericity. The optimized support bath led to an increased resolution – with printed filaments down to 20 μm in diameter –, and enabled direct printing of collagen, which usually requires some degree of modification or blending with other polymers to be successfully extruded [11, 25, 26]. Building on these results, a scaled-down beating ventricle model, a working tri-leaflet heart valve, and a perfusable multi-scale vasculature – replicated from MRI data – were successfully printed (Fig. 1h-k). Concurrently, *Skylar-Scott* et al. [18] developed the so-called sacrificial writing into functional tissue (SWIFT) technique, in which a support bath made of dense cellular spheroids sustains the printing of a gelatin sacrificial ink. Cellular spheroids from human embryonic or induced pluripotent stem cells (iPSCs) can be either undifferentiated embryoid bodies (EBs) or differentiated organoids (e.g. cardiac or cerebral). The final result is a branched hierarchical vascular network, successfully endothelialized with HUVECs, enclosed in a compacted tissue construct (Fig. 1l, m). The only limitation was that to maintain high fidelity the diameter of the sacrificial filaments needed to be ~ 400 μm, twice the size of the spheroids. While making a small step back in terms of printing resolution, using spheroids is a leap forward from the biological point of view, opening the way to vascularized constructs made from organoids and multicellular spheroids.

## Extrusion bioprinting: coaxial nozzles

A different way of using extrusion bioprinting to create hollow channels is integrating two or more coaxial nozzles, where an inner needle with a small diameter extrudes a crosslinker liquid solution (e.g. CaCl) while a concentric larger nozzle extrudes the crosslinkable hydrogel (e.g. alginate). The hydrogel reticulates while being printed, thus retaining its cylindrical hollow shape [9]. Several research papers exploited this technique [27–29], and *Jia* et al. showed good endothelialization of the hollow channels and a 21-days cell culture under perfusion [30]. More recently, *Shao* et al. obtained thicker vascularized constructs – up to 1 cm – and were able to combine three coaxial nozzles to yield multicellular tissue matrices [31]. While the main drawback of coaxial bioprinting is in creating bifurcations, it also shares a hurdle with the other extrusion printing techniques in creating hierarchical networks, since vessels are usually created by a single extrusion of filament.

## Stereolithography

Stereolithography, a maskless photolithography, uses light to crosslink a material with a resolution much higher than any extrusion technique, thus enabling the generation of intricate networks of channels with varying diameters [32, 33]. Customizing a stereolithography apparatus for tissue engineering (SLATE), *Grigoryan* et al. [34] produced alveolar models that were flexible enough to withstand cyclic ventilation of the hollow internal volumes, while ensuring constant perfusion of human red blood cells in the surrounding branching vascular network (Fig. 1n, o). Their airway-like models successfully oxygenated the perfused blood under cyclic ventilation. To further study the capabilities of their approach they also engineered a vascularized hepatic tissue construct (Fig. 1p). When implanted for 14 days in a rodent model of chronic liver injury, these constructs displayed albumin activity and promoted invasion of host blood cells. Moreover, in all their studies they used hydrophilic food additives as photoabsorbers, avoiding one of the main drawbacks of the commonly used photoabsorbers, toxicity.

## Conclusions

The vascularization of engineered tissues cannot be achieved using simple methods. The complexity of the tissue construct makes fabrication difficult both from the biological (need for at least two cell types: endothelial and tissue-specific) and the engineering point of view. It also requires long and complex protocols that need to be carefully crafted and standardized to obtain clinically viable and consistent products. A combination of different printing techniques might be the next step in the creation of multi-scale channels and hierarchical branched networks. The choice of materials also plays a pivotal role, usually being a tradeoff between ease of use and biocompatibility. While chemical functionalization and artificial polymers often improve the former, they often hinder the latter, which is usually guaranteed by the use of natural polymers or decellularized extracellular matrices. Several studies have already proven the effectiveness of both natural polymers and decellularized extracellular matrices for in vitro constructs [35, 36], but their use in bioprinting is still limited and requires highly engineered set-ups [24], mainly due to their viscosity and mechanical properties. Interesting results could also be obtained developing and using bioactive materials in combination with stem cells, as in the so-called 4D bioprinting, the printing of materials that can undergo conformational changes as a response to certain stimuli or to the action of cells [37]. Ultimately, surpassing these challenges will ensure the development of fast and consistent bioengineering strategies, scaling up the dimensions, and shifting from producing tissue patches to creating whole organs.
